# Supplementary material for: Esr1-Dependent Signaling and Transcriptional Maturation in the Medial Preoptic Area of the Hypothalamus Shapes the Development of Mating Behavior during Adolescence
Source: bioRxiv. 2025 Feb 26:2025.02.26.640339. Preprint. [Version 1] doi: 10.1101/2025.02.26.640339 (PMC11888408; doi:10.1101/2025.02.26.640339)
Supplement: 1 — Supplementary Fig. 1a: paired t-test. t(11)=−0.523 , p=0.612. Supplementary Fig. 1c: Two-way repeated measures ANOVA followed by multiple comparisons. ANOVA revealed main effect of age (F (2, 48) = 11.4416728, p<0.0001), main effect of group (F (1, 24) = 16.8147242, p<0.001) and interaction between group and age (F (2, 48) = 0.5084058, p=0.60). Tukey multi-comparison test was conducted. *p < 0.05. Supplementary Fig. 1d left: unpaired t-test. t(24)= −2.966, p=0.0067 Supplementary Fig. 1d right: unpaired t-test. t(15.466)= −3.7502, p= 0.0018 Supplementary Fig. 1e top: Body weight: Two-way repeated measures ANOVA. ANOVA revealed main effect of age (F (3.062, 73.49) = 51.24, p<0.0001), no effect of group (F (1, 24) = 1.366, p=0.2539) and no interaction between group and age (F (12, 288) = 0.2786, p=0.9922). Locomotion: unpaired t-test. t(24)=1.134, p=0.2682. Time investigating male: unpaired t-test. t(24)=0.2027, p=0.8411. Time investigating female: unpaired t-test. t(24)=1.499, p=0.1469. Social Preference: unpaired t-test. t(24)=0.8857, p=0.3846. Time in open arm: unpaired t-test. t(14)=0.2707, p=0.7906. Supplementary Fig. 1e bottom: Body weight: Two-way repeated measures ANOVA followed by multiple comparisons. ANOVA revealed main effect of age (F (3.182, 73.19) = 202.5, p<0.0001), no effect of group (F (1, 23) = 1.788, p=0.1942) and interaction between group and age (F (12, 276) = 3.375, p=0.0001). Holm-Sidak multi-comparison test was conducted. No significance was observed at any age between groups. Locomotion: unpaired t-test. t(23)=0.06150, p=0.9515. Time investigating male: unpaired t-test. t(23)=0.5738, p=0.5717. Time investigating female: unpaired t-test. t(23)=2.048, p=0.0521. Social Preference: unpaired t-test. t(23)=1.563, p=0.1317. Time in open arm: unpaired t-test. t(14)=0.4188, p=0.6817. Supplementary Fig. 1f top: Body weight: Two-way repeated measures ANOVA. ANOVA revealed main effect of age (F (4.942, 88.95) = 104.5, p<0.0001), no effect of group (F (1, [file NIHPP2025.02.26.640339V1-supplement-1.pdf]

bioRxiv preprint doi: https://doi.org/10.1101/2025.02.26.640339; this version posted February 26, 2025. The copyright holder for this preprint (which was not certified by peer review) is the author/funder, who has granted bioRxiv a license to display the preprint in perpetuity. It is made available under aCC-BY 4.0 International license.

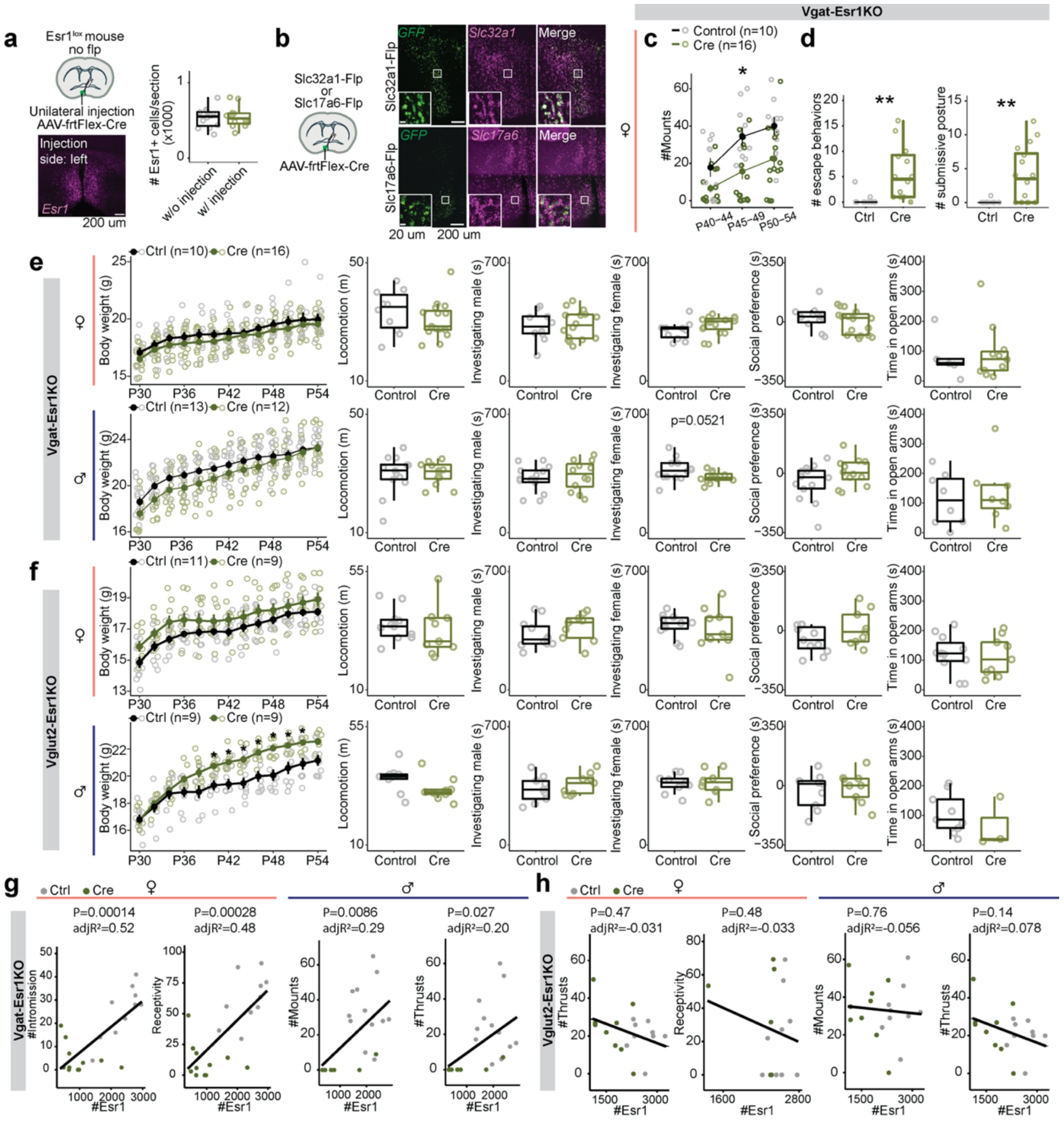

Supplementary Fig. 1: Supporting data for cell-type specific deletion of Esr1 and behavioral experiments, related to Fig. 1

a, Schematic illustrating unilateral injection of AAV-frtFlex-cre virus in the MPOA of Esr1$^{lox/lox}$ mice. Representative image of Esr1 expression in MPOA after a left hemisphere injection. Quantification of Esr1 in the MPOA. Note that this is a control experiment to test viral specificity, where mice did not express Flp-recombinase.

b, Schematic illustrating AAV-frtFlex-Cre injection in the MPOA of *Slc32a1*^{Flp} or *Slc17a6*^{Flp} mice. Representative images showing expression of *GFP* viral-reporter and *Slc32a1* (top) or *Slc17a6* (bottom) in the MPOA of *Slc32a1*^{Flp} (top) or *Slc17a6*^{Flp} (bottom) mice.

c-d, Quantitative comparisons of female mice: number of mounts received (**c**), escape attempts (**d**, left) and submissive postures (**d**, right) in *Slc32a1*^{Flp}::*Esr1*$^{lox/lox}$ mice.

e-f, From left to right, females (top) to males (bottom): Quantifications of body weight, locomotion, time investigating a male conspecific, time investigating a female conspecific, social preference in three chamber test, and time spent in the open arm of EPM of *Slc32a1*^{Flp}::*Esr1*$^{lox/lox}$ (**e**) or *Slc17a6*^{Flp}::*Esr1*$^{lox/lox}$ (**f**) mice.

bioRxiv preprint doi: https://doi.org/10.1101/2025.02.26.640339; this version posted February 26, 2025. The copyright holder for this preprint (which was not certified by peer review) is the author/funder, who has granted bioRxiv a license to display the preprint in perpetuity. It is made available under aCC-BY 4.0 International license.

g-h, Linear regression analysis between number of Esr1 cells and mating-related behavioral measurements in female (left) and male (right) *Slc32a1*^{Flp}::*Esr1*$^{lox/lox}$ (**g**) or *Slc17a6*^{Flp}::*Esr1*$^{lox/lox}$ (**h**) mice.

Line plots are shown in mean ± S.E.M and analyzed with a two-way repeated measures ANOVA followed by multiple comparisons. Box plots are shown with box (25%, median line, and 75%) and whiskers and analyzed with unpaired t-test. $^{**}p < 0.01$. $^{*}p < 0.05$. Statistical details in Methods.
aR^2: adjusted R squared; EPM: elevated plus maze.

bioRxiv preprint doi: https://doi.org/10.1101/2025.02.26.640339; this version posted February 26, 2025. The copyright holder for this preprint (which was not certified by peer review) is the author/funder, who has granted bioRxiv a license to display the preprint in perpetuity. It is made available under aCC-BY 4.0 International license.

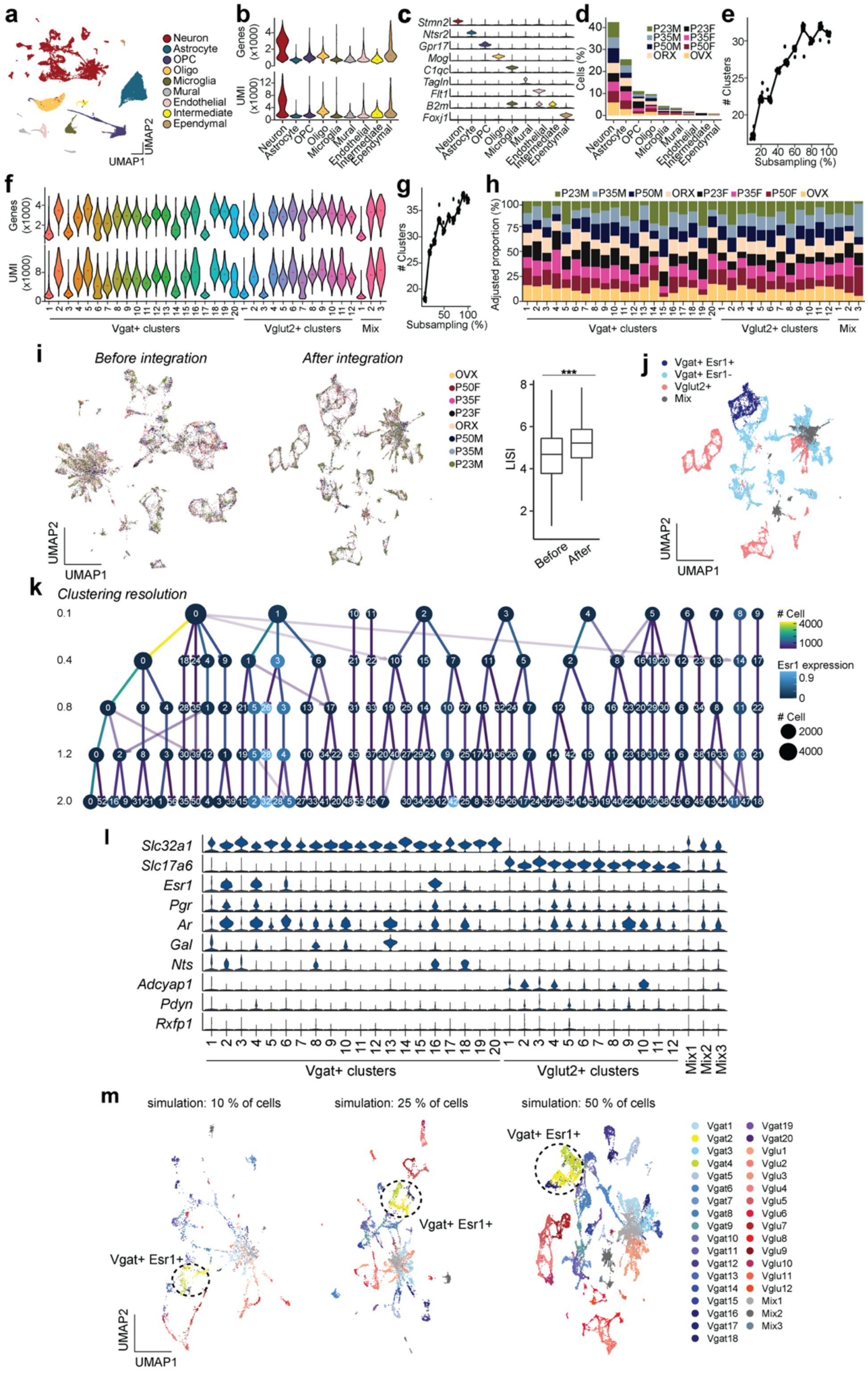

Supplementary Fig. 2: Supporting data for scRNAseq clustering, related to Fig. 2

a, Joint clustering of 58,921 cells from all groups (P23, P35, P50, GDX). UMAP plot is color coded by 9 cell category types, listed in legend.

b, Violin plots showing the gene (top) and UMI (bottom) distributions in each cell category type.

c, Violin plots of marker gene expression in each cell category type.

d, Proportion of cells represented in each cell category type from each group.

bioRxiv preprint doi: https://doi.org/10.1101/2025.02.26.640339; this version posted February 26, 2025. The copyright holder for this preprint (which was not certified by peer review) is the author/funder, who has granted bioRxiv a license to display the preprint in perpetuity. It is made available under aCC-BY 4.0 International license.

e, Graph representation of cluster number(s) from MPOA cell sub-sampling.
f, Violin plots showing the gene (top) and UMI (bottom) distributions in each neuronal cell type cluster.
g, Graph representation of cluster number(s) from MPOA neuron sub-sampling.
h, Proportion of cells represented in each neuron cluster from each group. All neuronal cell types were present irrespective of age, sex, and hormonal states.
i, UMAP clustering without integration (left) and then joint clustering (right) of neuronal cells from all groups. UMAP plot is color coded by experimental group. Boxplot of LISI (Local Inverse Simpson's Index, or cell "mixing" index) was computed to assess integration performance. Note that the theoretical maximum of LISI score is 8.
j, UMAP plot with color code for major neuronal types: Salmon = Vglut2+, Blue = Vgat+Esr1-, Purple = Vgat+Esr1+, Grey = Mixed.
k, Cluster tree illustrating the lineage of clusters at each clustering resolution. *Esr1* expression and the number of cells in each cluster are represented by the color and the size of the dot, respectively. Number of cells diverging from a node is represented by the color of the connecting line.
l, Violin plots showing normalized expression values of *Slc32a1*, *Slc17a6*, several steroid hormone receptor genes, and canonical marker genes in the MPOA at each cluster.
m, *In-silico* analysis generated UMAP plots, using simulated subsets of data. UMAP plots are color coded by neuronal types. Vgat+Esr1+ clusters are highlighted.

Box plots are shown with box (25%, median line, and 75%) and whiskers and analyzed with Wilcoxon rank-sum test. ***$p < 0.001$. Statistical details in Methods.
UMIs: unique molecular identifiers; GDX: gonadectomy; OVX: ovariectomy; ORX: orchiectomy.

bioRxiv preprint doi: https://doi.org/10.1101/2025.02.26.640339; this version posted February 26, 2025. The copyright holder for this preprint (which was not certified by peer review) is the author/funder, who has granted bioRxiv a license to display the preprint in perpetuity. It is made available under aCC-BY 4.0 International license.

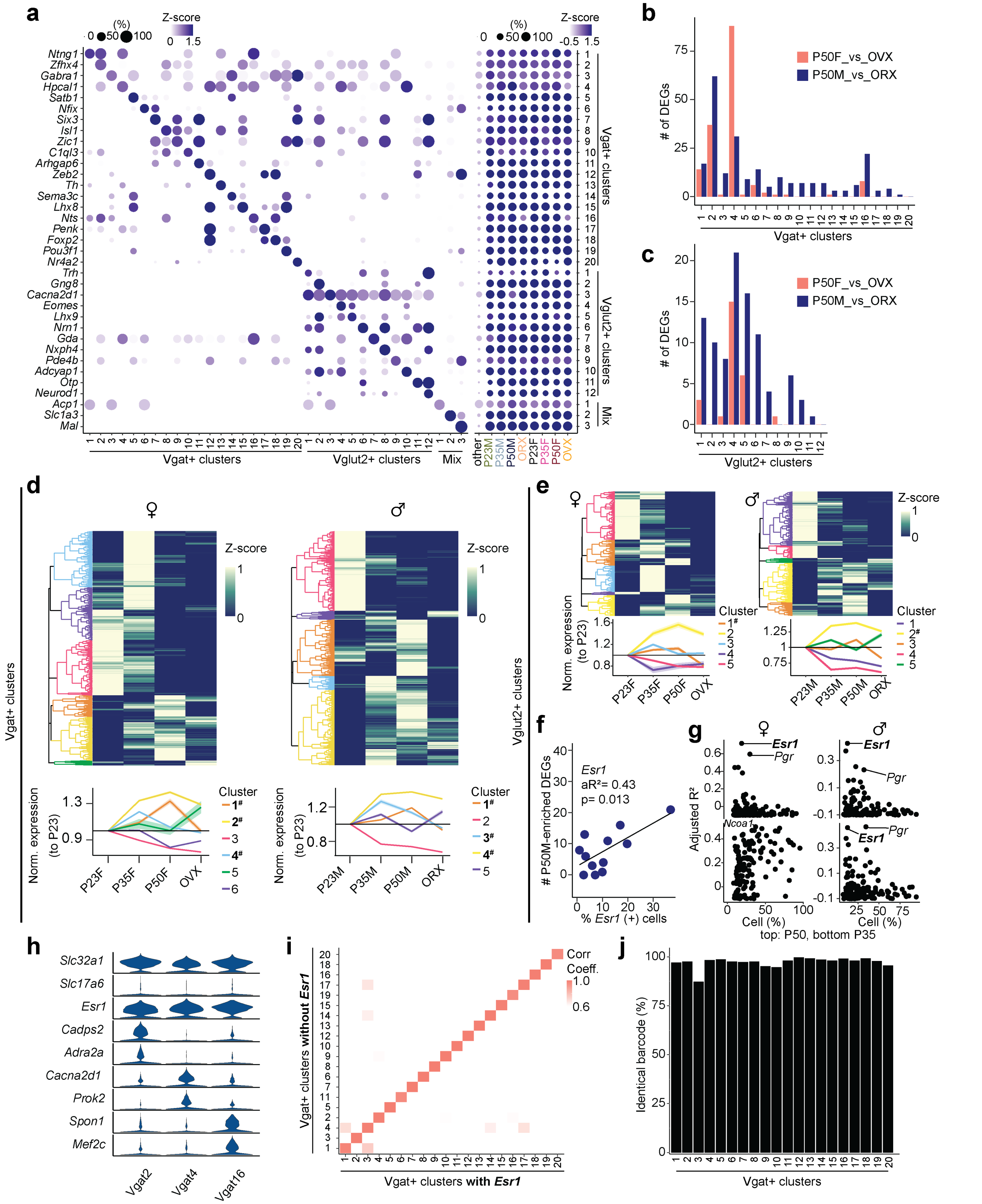

Supplementary Fig. 3: Supporting data for scRNAseq experiments, related to Fig. 2

a, Dot plot illustrating scaled expression (z-score, color intensity) and percentage of expressing cells (dot size) for a list of marker genes (y-axis) in each cluster (x-axis). On the left y-axis each clusters gene expression score is presented for each group: P23, P35, P50, and GDX (females and males).

bioRxiv preprint doi: https://doi.org/10.1101/2025.02.26.640339; this version posted February 26, 2025. The copyright holder for this preprint (which was not certified by peer review) is the author/funder, who has granted bioRxiv a license to display the preprint in perpetuity. It is made available under aCC-BY 4.0 International license.

b, c, Bar plot to show the difference in number of DEGs between P50 and GDX groups (females: salmon; males: blue) in each Vgat+ cluster (**b**) and Vglut2+ cluster (**c**).
d, e, Heatmaps with hierarchical clustering dendrograms (top) and line graphs (bottom) to demonstrate gene expression patterns within a cluster relative to other clusters for each group: P23, P35, P50, GDX in females (left) and males (right). Clusters are denoted by color. Cluster numbers that are bolded and marked with a hashtag (#) show higher expression at P35 and P50 than P23 and GDX. Z-scored gene expression is denoted by a separate color intensity scale. Line graph data shows normalized expression of each cluster relative to P23 expression. Vgat+ clusters (**d**); Vglut2+ clusters (**e**).
f, Linear regression analysis between percentage of *Esr1*-expressing cells and number of P50M-enriched DEGs compared to GDX for each Vglut2+ cluster (dot).
g, Scatter plots showing aR^2 values of hormone receptor genes (dots) in females (left) and males (right) comparing the percentage of percent expressing cells to the number of DEGs in comparison with GDX samples at each Vglut2+ cluster for P50-enriched (top) and P35-enriched (bottom) DEGs.
h, Violin plots showing the normalized expression values of *Slc32a1*, *Slc17a6*, *Esr1,* and several subtype-specific genes in the MPOA for each Vgat+Esr1+ cluster (Vgat 2, 4, 16).
i, Heatmap illustrating the Pearson correlation coefficient between Vgat+ clusters including or excluding *Esr1* expression data. Near identical clusters were detected irrespective of *Esr1* expression.
j, Bar plot showing the percent of identical cell barcodes within each Vgat+ cluster comparing inclusion and exclusion of *Esr1* expression data. Near identical clusters were detected irrespective of *Esr1* expression.

Line graphs include standard error and were analyzed using one-way repeated-measures ANOVA followed by multiple comparisons. Statistical details in Methods.
GDX: gonadectomy; OVX: ovariectomy; ORX: orchiectomy; aR^2: adjusted R squared; DEG: differentially expressed gene.

bioRxiv preprint doi: https://doi.org/10.1101/2025.02.26.640339; this version posted February 26, 2025. The copyright holder for this preprint (which was not certified by peer review) is the author/funder, who has granted bioRxiv a license to display the preprint in perpetuity. It is made available under aCC-BY 4.0 International license.

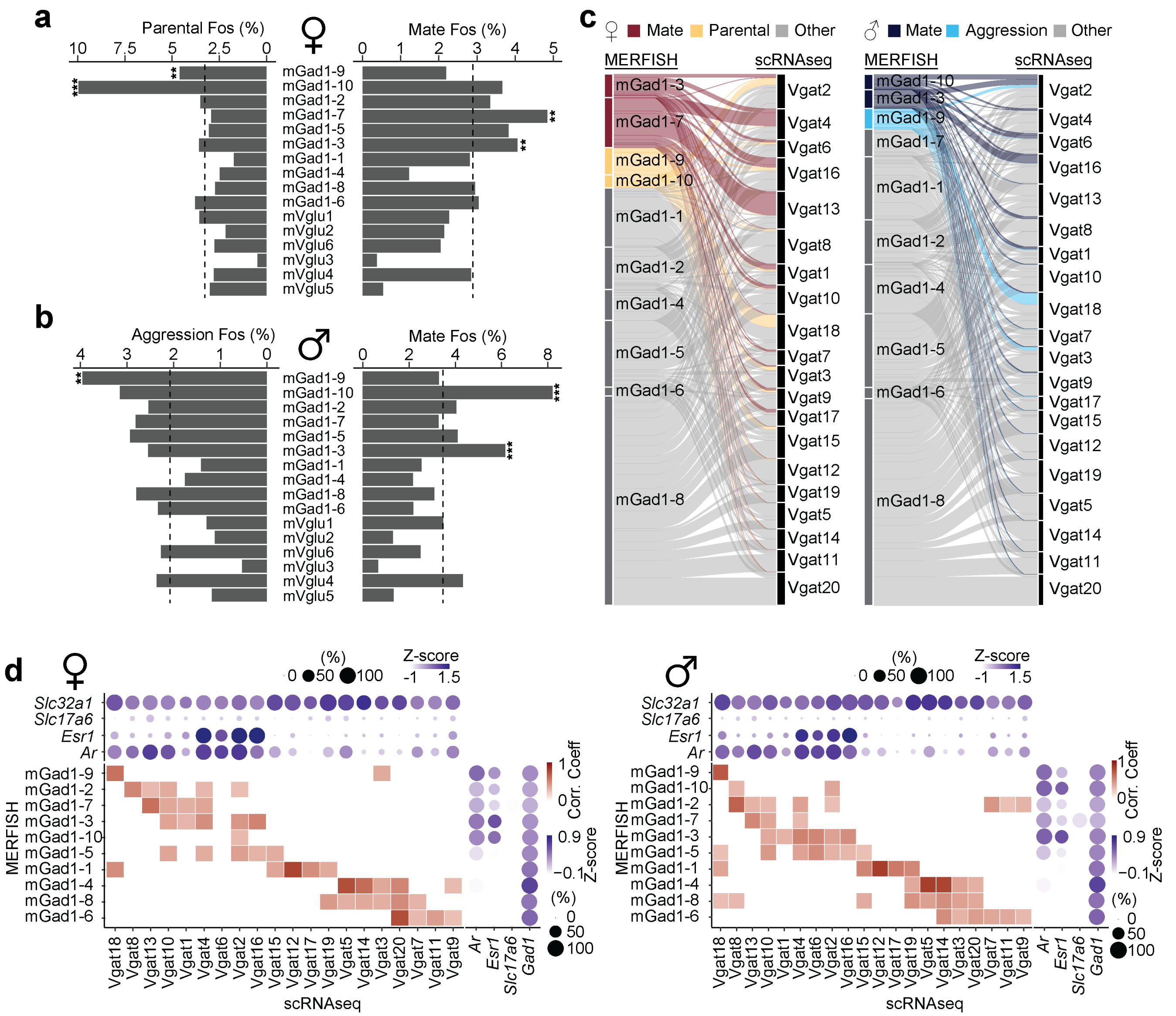

Supplementary Fig. 4: Supporting data for scRNAseq and MERFISH analysis, related to Fig. 2

a, b, Percentage of *Fos* positive cells associated with female parental and mating behaviors (**a**) and male aggression and mating behaviors (**b**) from MERFISH cell clusters (Moffitt et al., 2018). Dashed lines indicate the overall mean percentage. Each cluster was compared to the mean using a Fisher's exact test. $^{**}p < 0.01$, $^{***}p < 0.001$. Statistical details in Methods.

c, Alluvial plots showing the correspondence between MERFISH and scRNAseq clusters color coded by behavior.

d, Heatmaps and dot plots showing both Pearson correlation coefficients of MERFISH + scRNAseq GABAergic (mGad; Vgat) clusters and their expression of selected genes: *Slc32a1*, *Slc17a6*, *Esr1*, *Ar,* or *Gad1* in the scRNAseq dataset (top) and the MERFISH dataset (right). Increased color intensity corresponds to greater values. Dot size represents percentage of cells expressing the corresponding gene. Left: females; right: males.

bioRxiv preprint doi: https://doi.org/10.1101/2025.02.26.640339; this version posted February 26, 2025. The copyright holder for this preprint (which was not certified by peer review) is the author/funder, who has granted bioRxiv a license to display the preprint in perpetuity. It is made available under aCC-BY 4.0 International license.

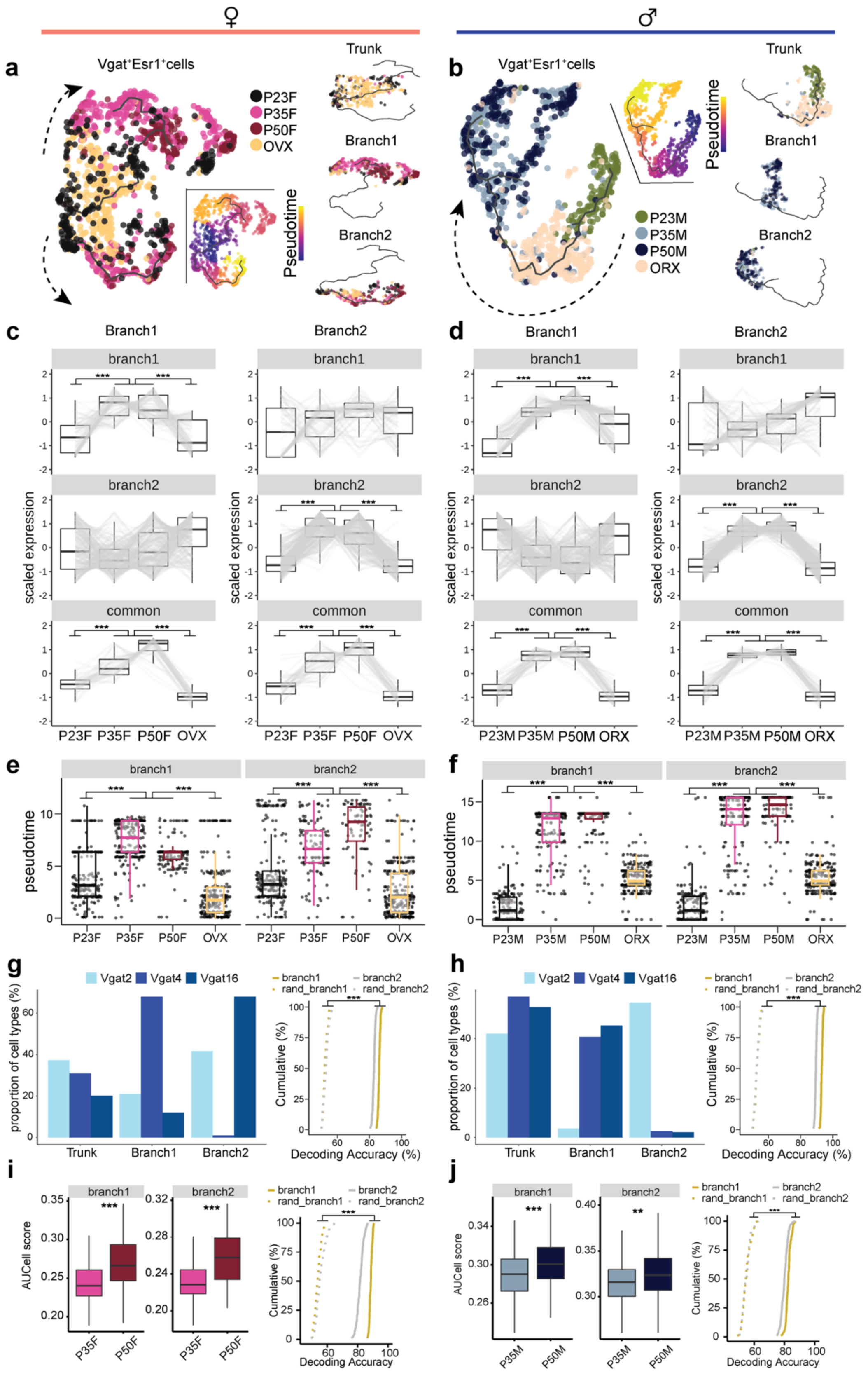

Supplementary Fig. 5: Supporting data for transcriptional trajectory branches from scRNAseq data, related to Fig. 3

a, b, UMAP visualization of Vgat+Esr1+ cells and their transcriptional trajectories depicted by a solid black line. Vgat+Esr1+ cells are color coded by group (left) and pseudotime (right), where progression of

bioRxiv preprint doi: https://doi.org/10.1101/2025.02.26.640339; this version posted February 26, 2025. The copyright holder for this preprint (which was not certified by peer review) is the author/funder, who has granted bioRxiv a license to display the preprint in perpetuity. It is made available under aCC-BY 4.0 International license.

time is delineated from dark to bright coloring. Dashed arrows indicate the direction of transcriptional progression. Smaller UMAPs on the right visually isolate the trunk and two identified branch trajectories. **a**: females; **b**: males.

c, d, Box plots show scaled gene expression in Vgat+Esr1+ cells from Branch 1 (left column) and Branch 2 (right column) trajectories for Branch 1-specific genes (top row), Branch 2-specific genes (middle row), and Branch-common genes (bottom row) across all groups.

e, f, Box plots show pseudotime score for Branch 1 (left column) and Branch 2 (right column) trajectories across groups in females (**e**) and males (**f**).

g, h, Bar plots represent the percentage of Vgat+Esr1+ cells that belong to cluster Vgat 2, 4, and 16 in the trunk and branches. Cumulative distributions for decoding accuracy between more mature (P35, P50) and immature (P23, GDX) groups using Branch 1 (yellow) or Branch 2 (grey) -specific genes compared to shuffled data (dashed line). **g:** females; **h:** males.

i, j, Box plots of HA-DEG aggregate expression (AUCell score) separated by branch in P35 and P50. Cumulative distributions for decoding accuracy between P35 and using Branch 1 (yellow) or Branch 2 (grey) -specific genes compared to shuffled data (dashed line). **i:** females; **j:** males.

Box plots are shown with box (25%, median line, and 75%) and whiskers and analyzed using Kruskal-Wallis H test followed by multiple comparisons test. p-values were Bonferroni corrected. Cumulative distributions were analyzed with unpaired t-tests for each branch. $^{**}p < 0.01$, $^{***}p < 0.001$. Statistical details in Methods.

HA-DEG: hormone-associated differentially expressed gene; GDX: gonadectomy; OVX: ovariectomy; ORX: orchiectomy.

bioRxiv preprint doi: https://doi.org/10.1101/2025.02.26.640339; this version posted February 26, 2025. The copyright holder for this preprint (which was not certified by peer review) is the author/funder, who has granted bioRxiv a license to display the preprint in perpetuity. It is made available under aCC-BY 4.0 International license.

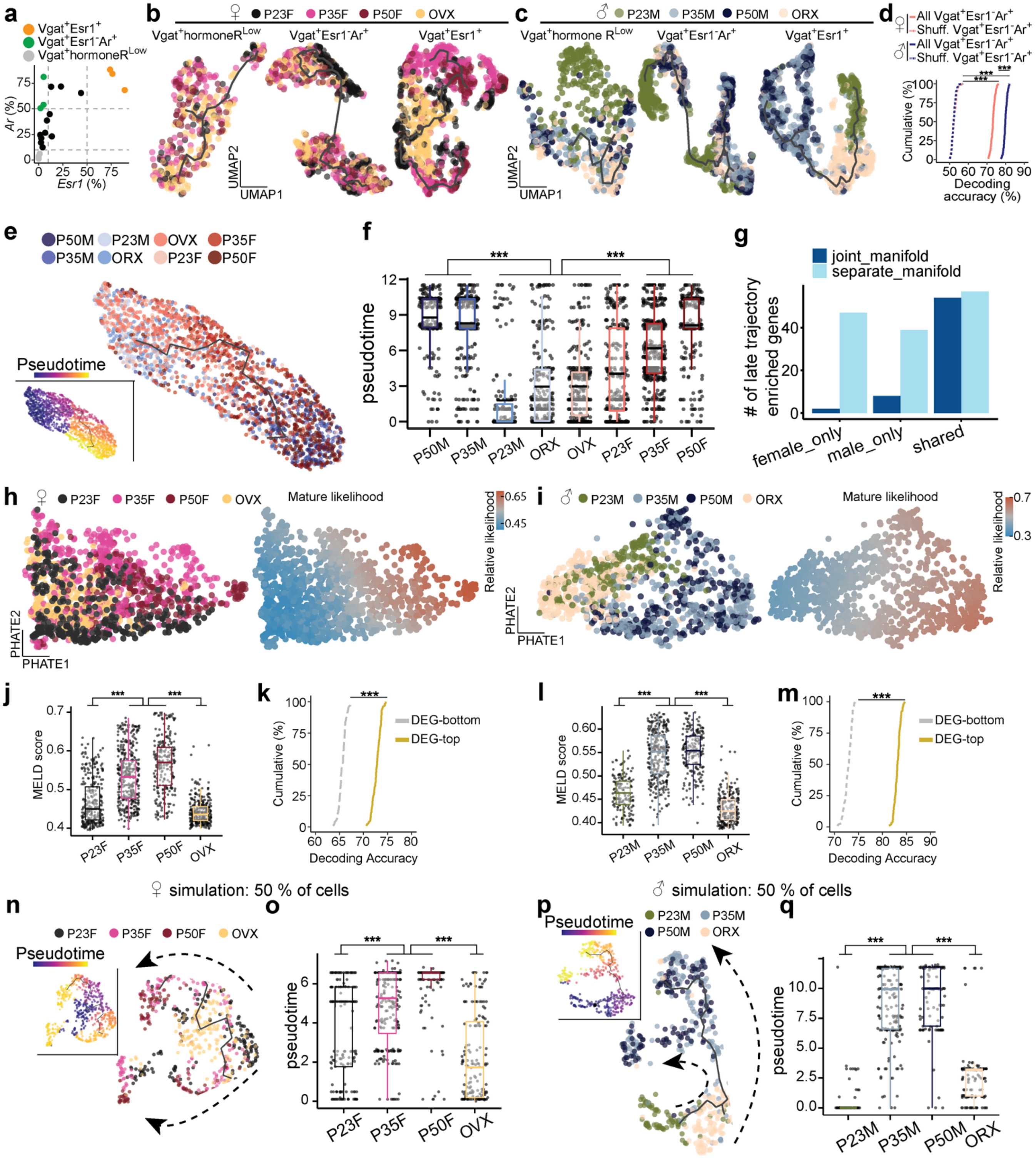

Supplementary Fig. 6: Supporting data for scRNAseq trajectory analysis, related to Fig. 3

a, Scatter plot showing the percentage of *Esr1* and/or *Ar* expressing cells in each Vgat+ cluster (dot). **b,c,** UMAP visualization of transcriptional trajectories (black line) color coded by experimental group in Vgat+hormoneR^{Low} (left), Vgat+Esr1-Ar+ (middle), and Vgat+Esr1+ (right) in females (**b**) and males (**c**). **d,** Cumulative distributions of decoding accuracy by SVM classification between mature groups (P50, P35) and immature groups (P23, GDX) using expression data from Vgat+Esr1-Ar+ (salmon: female, blue: male, solid line), or shuffled data (dashed line).

bioRxiv preprint doi: https://doi.org/10.1101/2025.02.26.640339; this version posted February 26, 2025. The copyright holder for this preprint (which was not certified by peer review) is the author/funder, who has granted bioRxiv a license to display the preprint in perpetuity. It is made available under aCC-BY 4.0 International license.

e, UMAP visualization of Vgat+Esr1+ cells combining males and females, depicting their transcriptional trajectory denoted by a solid black line. Vgat+Esr1+ cells are color coded by group (right) and pseudotime (left), where progression of time is delineated from dark to bright coloring.
f, Box plot of pseudotime score from combined male and female UMAP (**e**) separated by group.
g, Bar plot showing the numbers of sex-specific and shared genes enriched in later trajectory, which were identified using a separate manifold for each sex or a joint manifold. Majority of sex-specific gene programs were not identified in joint manifold analysis.
h,i, PHATE visualization of transcriptional progression in Vgat+Esr1+ cells (dot) color coded by group (left) and relative mature likelihood (right) in females (**h**) and males (**i**).
j, l, Box plot showing MELD score (mature likelihood) of Vgat+Esr1+ cells in females (**j**) and males (**l**).
k,m, Cumulative distributions of decoding accuracy between mature groups (P50, P35) and immature groups (P23, GDX) using highest expressing (yellow, used in the HCR experiments) or lowest expressing (grey) HA-DEGs in females (**k**) and males (**m**).
n,p, *In silico* simulation using a 50% subset of the data. UMAP visualization of transcriptional trajectory (black line) and cells (dots) color coded by group (right) and pseudotime (left) in female (**n**) and male (**p**) Vgat+Esr1+ cells. Dashed arrows indicate the direction of transcriptional progression.
o, q, *In silico* simulation analysis using a 50% subset of the data. Box plots show pseudotime values assigned to each Vgat+Esr1+ cell across groups in females (**o**) and males (**q**).
o, UMAP visualization of transcriptional trajectory (black line) and cells (dots) color-coded by group (right) or pseudotime (left) in Vgat+Esr1+populations, including both sexes.

Box plots are shown with box (25%, median line, and 75%) and whiskers and analyzed using Kruskal-Wallis H test followed by multiple comparisons test. p-values were Bonferroni corrected. Cumulative distributions were analyzed with unpaired t-tests. ***$p < 0.001$. Statistical details in Methods.
SVM: support vector machine.

bioRxiv preprint doi: https://doi.org/10.1101/2025.02.26.640339; this version posted February 26, 2025. The copyright holder for this preprint (which was not certified by peer review) is the author/funder, who has granted bioRxiv a license to display the preprint in perpetuity. It is made available under aCC-BY 4.0 International license.

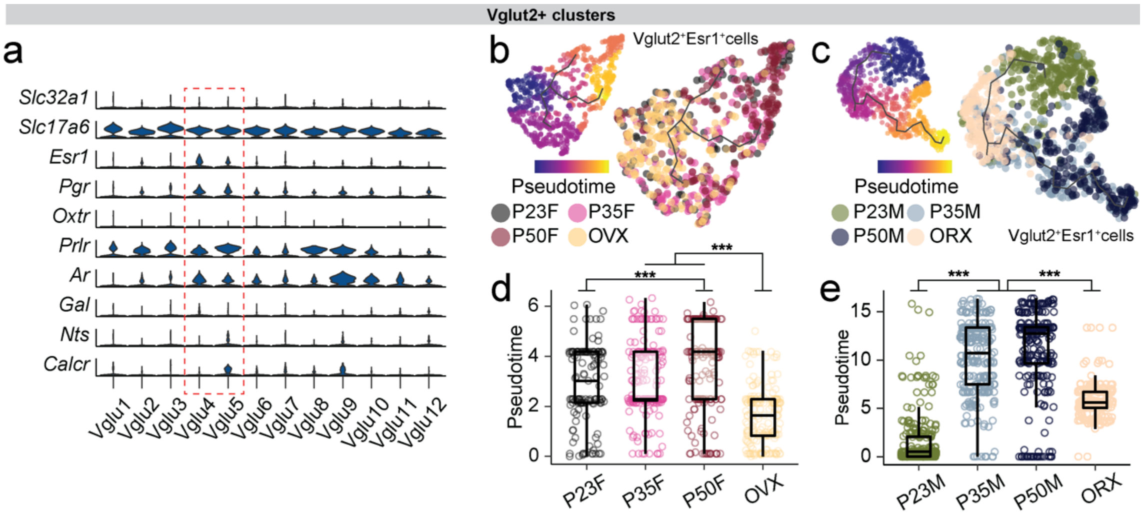

Supplementary Fig. 7: Supporting information for trajectory analysis on scRNAseq data, related to Fig. 3

a, Violin plots show normalized expression values of *Slc32a1*, *Slc17a6*, several steroid hormone receptor genes, and canonical marker genes in the MPOA at each Vglut2+ cluster. Vglut2+Esr1+ clusters are highlighted.

b, c, UMAP visualization of transcriptional trajectory (black line) and cells (dots) color coded by pseudotime (left) or group (right) in female (**b**) and male (**c**) Vglut2+Esr1+ populations.

d, e, Box plot showing pseudotime of Vglut2+Esr1+ cells in females (**d**) and males (**e**).

Box plots are shown with box (25%, median line, and 75%) and whiskers and analyzed using Kruskal-Wallis H test followed by multiple comparisons test. p-values were Bonferroni corrected. ***$p < 0.001$. Statistical details in Methods.

bioRxiv preprint doi: https://doi.org/10.1101/2025.02.26.640339; this version posted February 26, 2025. The copyright holder for this preprint (which was not certified by peer review) is the author/funder, who has granted bioRxiv a license to display the preprint in perpetuity. It is made available under aCC-BY 4.0 International license.

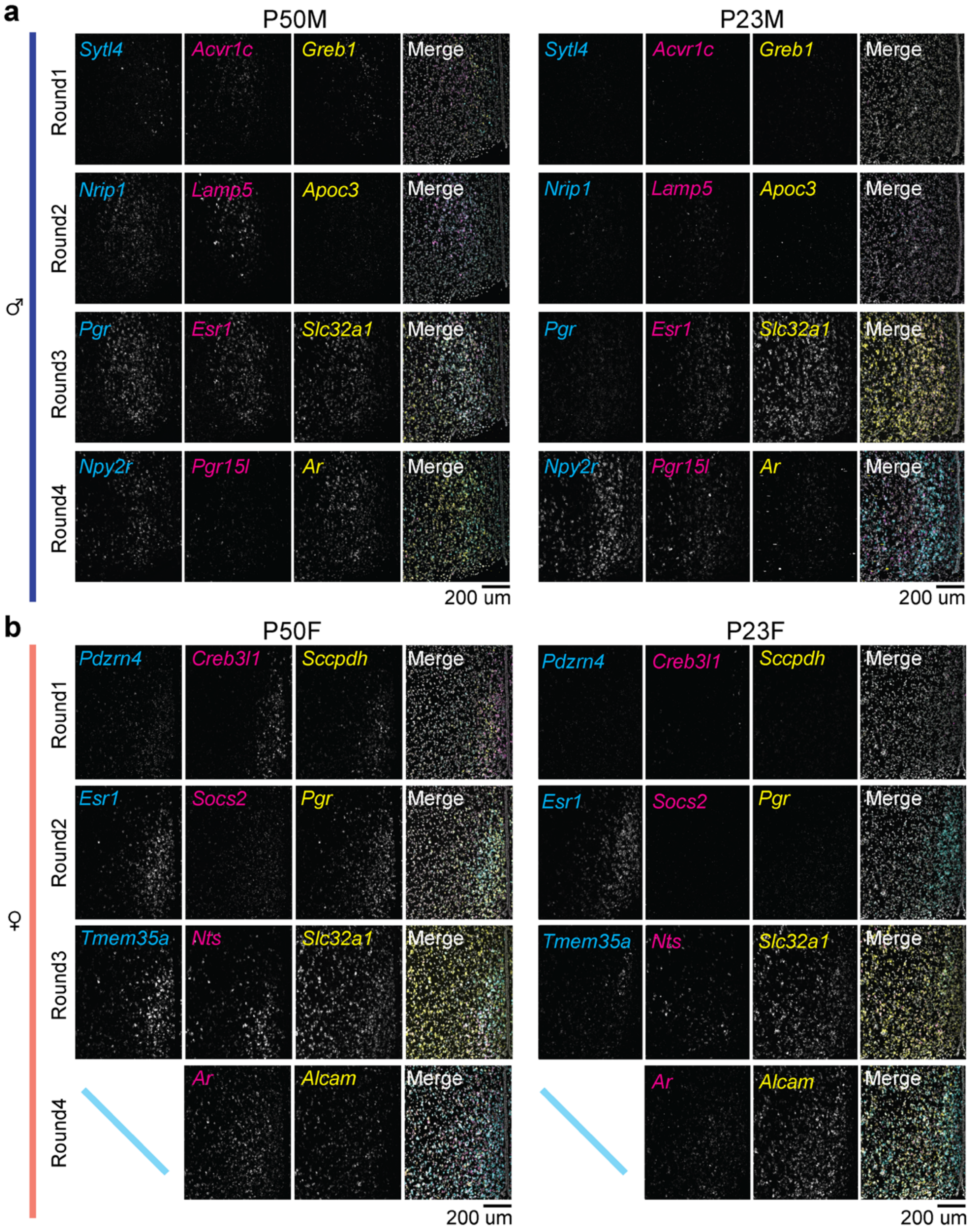

Supplementary Fig. 8: Supporting data for HM-HCR FISH, related to Fig. 4

a, b, Representative images showing detected genes in 4 iterative rounds of HM-HCR FISH from the MPOA at P50 (left) and P23 (right). **a**: males; **b**: females. Scale bars: 200 µm.

bioRxiv preprint doi: https://doi.org/10.1101/2025.02.26.640339; this version posted February 26, 2025. The copyright holder for this preprint (which was not certified by peer review) is the author/funder, who has granted bioRxiv a license to display the preprint in perpetuity. It is made available under aCC-BY 4.0 International license.

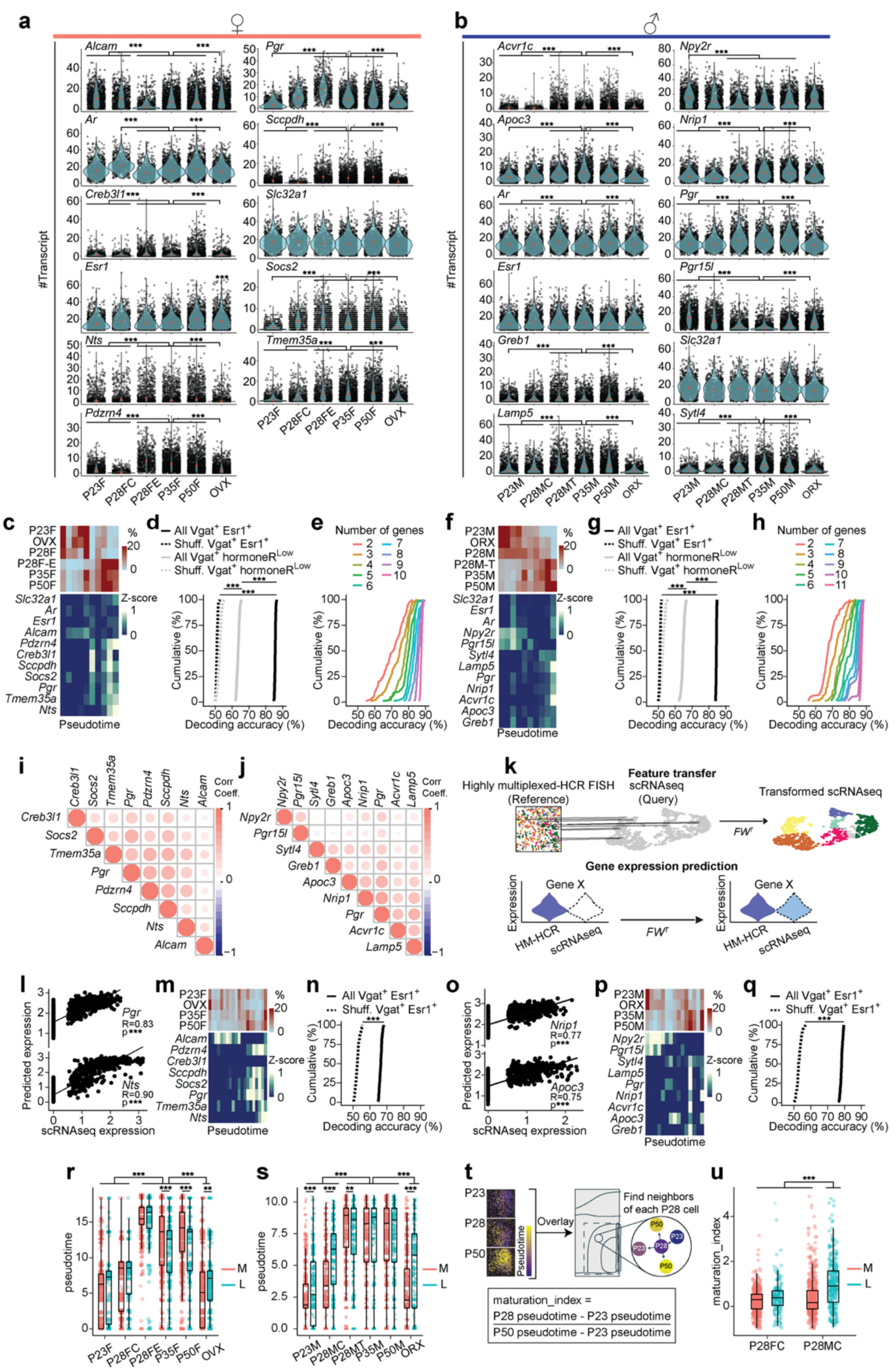

Supplementary Fig. 9: Supporting data for HM-HCR FISH experiments related to Fig. 4.

a, b, Violin plots showing the expression values of individual genes across experimental groups in MPOA Vgat+Esr1+ cells. **a**: females; **b**: males.

c, f, Heatmaps showing the percentage of Vgat+Esr1+ cells (top) and scaled gene expression (bottom) across pseudotime. **c**: females; **f**: males.

d, g, Cumulative distributions of decoding accuracy between mature groups (P50, P35, hormone-supplemented P28) and immature groups (P23, GDX, P28 control) using expression data from

bioRxiv preprint doi: https://doi.org/10.1101/2025.02.26.640339; this version posted February 26, 2025. The copyright holder for this preprint (which was not certified by peer review) is the author/funder, who has granted bioRxiv a license to display the preprint in perpetuity. It is made available under aCC-BY 4.0 International license.

Vgat+Esr1+ (black, solid line), Vgat+hormoneR^{Low} (grey, solid line), and shuffled data (dashed lines). **d**: females; **g**: males.
e, h, Cumulative distributions of decoding accuracy between mature (P50, P35, hormone-supplemented P28) and immature (P23, GDX, P28 control) groups using subsets of Vgat+Esr1+ gene expression data, color coded by number of genes used for decoding. **e**: females; **h**: males.
i**, j,** Correlograms of Pearson correlation coefficients between all pairs of DEGs in MPOA Vgat+Esr1+ cells. **i**: females; **j**: males.
k, Schematic illustrating integrative analysis between HM-HCR FISH and scRNAseq datasets to predict scRNAseq gene expression.
l, o, Scatter plots showing the correlated expressions of real and predicted scRNAseq data. **l**: females; **o**: males.
m, p, Heatmaps showing the percentage of Vgat+Esr1+ cells (top) and scaled predicted gene expression (bottom) across pseudotime. **m**: females; **p**: males.
n, p, Cumulative distributions of decoding accuracy between mature groups (P50, P35) and immature groups (P23, GDX) using predicted expression data in females (**n**) and males (**p**).
r-u, Quantitative analyses of pseudotime in space, corresponding to **Fig. 4f, g**.
r, s, Boxplots showing pseudotime of Vgat+Esr1+ cells in the medial and lateral MPOA of females (**r**) and males (**s**).
t, Schematic illustrating the quantification of sex differences across the transcriptional progression rate. Neighborhood analysis was performed in physical space to compute the maturation index of cells from P28 control groups.
u, Box plot of maturation index data from Vgat+Esr1+ cells in the medial (M) and lateral (L) MPOA of P28 female and male controls. At P28, male mice showed an early onset of transcriptional progression in the lateral part of the MPOA.

Violin plots are outlined with a distribution line, individual dots represent each cell, and red dot indicates the median. Violin plots are analyzed with Wilcoxon rank-sum test. Box plots are shown with box (25%, median line, and 75%) and whiskers and analyzed using Kruskal-Wallis H test followed by multiple comparisons test. p-values were Bonferroni corrected. Cumulative distributions were analyzed with unpaired t-tests. ***$p < 0.001$. Statistical details in Methods.
R: Pearson correlation coefficient; M: medial MPOA; L: lateral MPOA; P28FC: P28 female control; P28FE: P28 females with estrogen treatment; P28MC: P28 male control; P28MT: P28 males with testosterone treatment; GDX: gonadectomy; OVX: ovariectomy; ORX: orchiectomy.

bioRxiv preprint doi: https://doi.org/10.1101/2025.02.26.640339; this version posted February 26, 2025. The copyright holder for this preprint (which was not certified by peer review) is the author/funder, who has granted bioRxiv a license to display the preprint in perpetuity. It is made available under aCC-BY 4.0 International license.

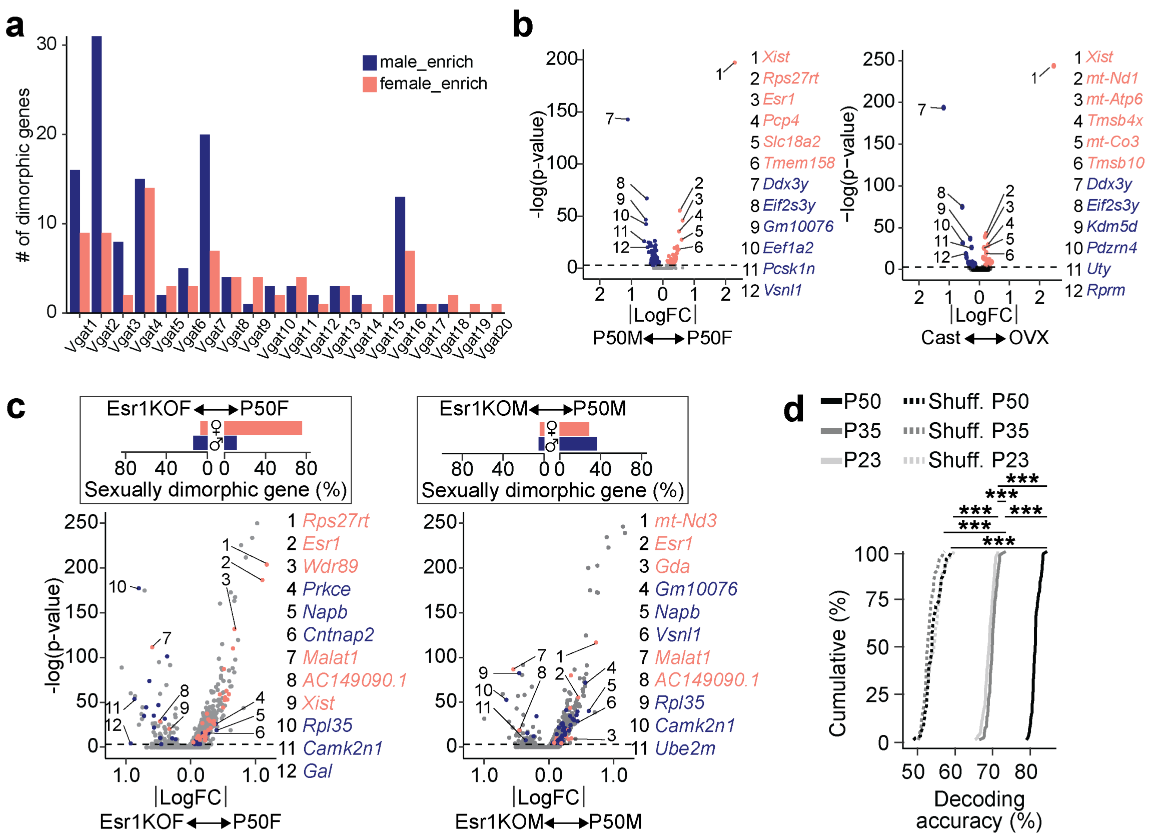

Supplementary Fig. 10: Supporting data for sexual dimorphism analysis, related to Fig. 5-7

a, Bar plot showing the number of sexually dimorphic genes at each Vgat+ cluster comparing female (salmon) and male (blue) groups at P50.

b, Volcano plot comparing P50 female (salmon) and male (blue) gene expression in Vgat+Esr1+ cells (left). Dimorphic genes are highlighted and indicated numerically. This is the same panel shown in **Fig. 5e** and reused here to facilitate the comparison with GDX data shown on the right. Volcano plot comparing OVX (salmon) and ORX (blue) gene expression in Vgat+Esr1+ cells. Dimorphic genes are highlighted and indicated numerically.

c, Volcano plots comparing intact P50 to Esr1KO P50 gene expression in Vgat+Esr1+ cells. The percent of sexually dimorphic genes (P50F-enriched genes: salmon, P50M-enriched genes: blue) present in intact P50 and Esr1KOs are quantified in the bar plot (top). Left: females; right: males.

d, Cumulative distributions of decoding accuracy between males and females (P50: black, P35: dark grey, P23: light grey, shuffled: dashed).

Cumulative distributions were analyzed with one-way ANOVA followed by multiple comparisons. Details in Methods. ***p < 0.001. Statistical details in Methods.

GDX: gonadectomy; OVX: ovariectomy; ORX: orchiectomy; LogFC: Log fold change.

bioRxiv preprint doi: https://doi.org/10.1101/2025.02.26.640339; this version posted February 26, 2025. The copyright holder for this preprint (which was not certified by peer review) is the author/funder, who has granted bioRxiv a license to display the preprint in perpetuity. It is made available under aCC-BY 4.0 International license.

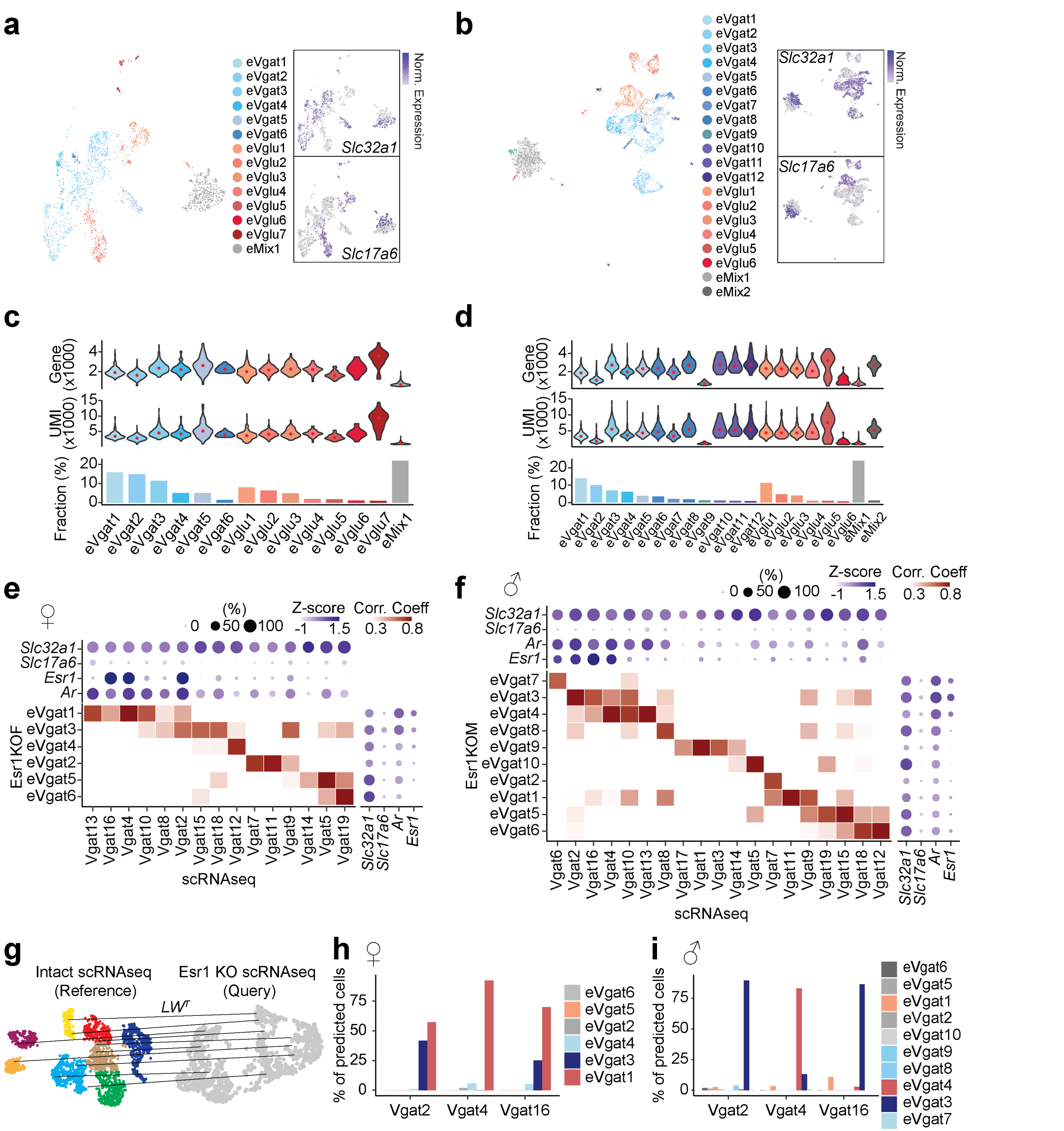

Supplementary Fig. 11: Supporting data for trajectory and gene regulatory network analysis followed by cell type specific deletion of *Esr1*, related to Fig. 7

a, b, UMAP visualization of neuronal clusters in Esr1KOF (**a**, left) and Esr1KOM (**b**, left). Feature plots showing normalized expression of *Slc32a1* and *Slc17a6* in Esr1KOF (**a**, right) and Esr1KOM (**b**, right).

c, d, Violin plots showing gene (top) and UMI (middle) distributions in each neuronal cell type. Bar graphs show the fraction representation of each neuronal cluster in Esr1KOF (**c**, bottom) and Esr1KOM (**d**, bottom).

e, f, Heatmaps illustrating Pearson correlation coefficient (red) between Vgat+ clusters of Esr1KO and intact groups. Dot plots are attached illustrating scaled expression (color intensity) and the percentage of expressing cells (dot size) of *Slc32a1*, *Slc17a6*, *Esr1,* and *Ar* in intact (top) and Esr1KOs (right side) in females (**e**) and males (**f**).

bioRxiv preprint doi: https://doi.org/10.1101/2025.02.26.640339; this version posted February 26, 2025. The copyright holder for this preprint (which was not certified by peer review) is the author/funder, who has granted bioRxiv a license to display the preprint in perpetuity. It is made available under aCC-BY 4.0 International license.

g, Schematic illustrating integrative analysis to establish correspondence between cell types of Esr1KO data and the reference scRNAseq data.
h, i, Bar graphs showing the correspondence between Esr1KO and the reference Vgat+Esr1+ clusters in females (**h**) and males (**i**) using the label transfer technique (details in Methods).

Violin plots are outlined with a distribution line and red dot indicates the median.

bioRxiv preprint doi: https://doi.org/10.1101/2025.02.26.640339; this version posted February 26, 2025. The copyright holder for this preprint (which was not certified by peer review) is the author/funder, who has granted bioRxiv a license to display the preprint in perpetuity. It is made available under aCC-BY 4.0 International license.

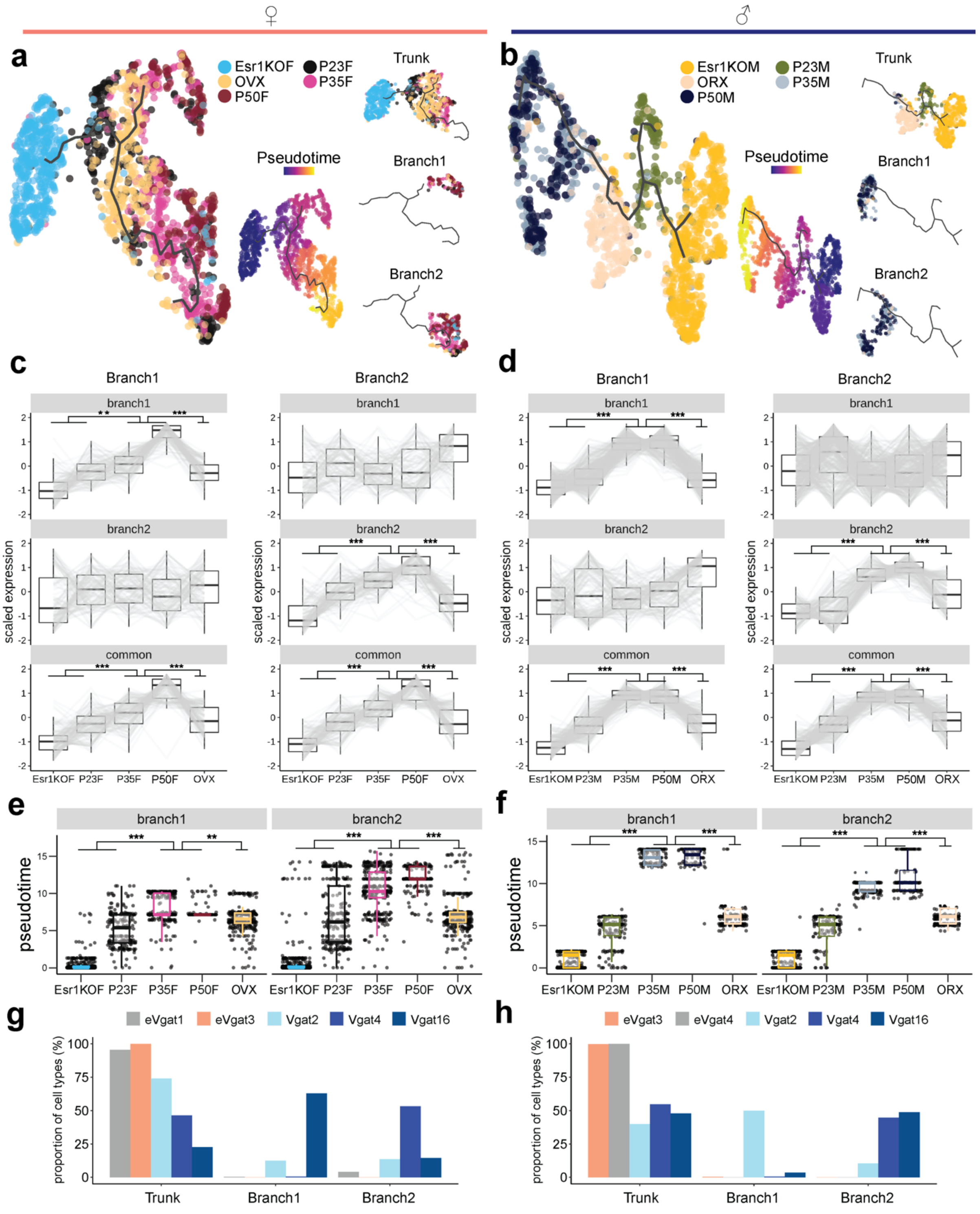

Supplementary Fig. 12: Supporting data for trajectory analysis followed by cell type specific deletion of *Esr1*, related to Fig. 7

a,b, UMAP visualization of transcriptional trajectories (black line) and cells (dots) color coded by experimental group (left) and pseudotime (inset) in Vgat+Esr1+ cells. UMAPs on the right visualize where cells from the branches and trunk lie on the trajectories. **a:** females; **b:** males.

c, d, Box plots show scaled gene expression in Vgat+Esr1+ cells from Branch 1 (left column) and Branch 2 (right column) trajectories for Branch 1-specific genes (top row), Branch 2-specific genes (middle row), and Branch-common genes (bottom row) across all groups. **c:** females; **d:** males.

bioRxiv preprint doi: https://doi.org/10.1101/2025.02.26.640339; this version posted February 26, 2025. The copyright holder for this preprint (which was not certified by peer review) is the author/funder, who has granted bioRxiv a license to display the preprint in perpetuity. It is made available under aCC-BY 4.0 International license.

e, f, Box plots show pseudotime score for Branch 1 (left column) and Branch 2 (right column) trajectories across groups in females (**e**) and males (**f**).
g,h, Bar plot of Vgat+Esr1+ cluster (eVgat for Esr1KOs) proportion of cells that reside in the trunk, branch1, or branch2. **g:** females; **h:** males.

Box plots are shown with box (25%, median line, and 75%) and whiskers and analyzed using Kruskal-Wallis H test followed by multiple comparisons test. p-values were Bonferroni corrected. ***$p < 0.001$, **$p < 0.01$. Statistical details in Methods.
GDX: gonadectomy; OVX: ovariectomy; ORX: orchiectomy; KO: knockout.

bioRxiv preprint doi: https://doi.org/10.1101/2025.02.26.640339; this version posted February 26, 2025. The copyright holder for this preprint (which was not certified by peer review) is the author/funder, who has granted bioRxiv a license to display the preprint in perpetuity. It is made available under aCC-BY 4.0 International license.

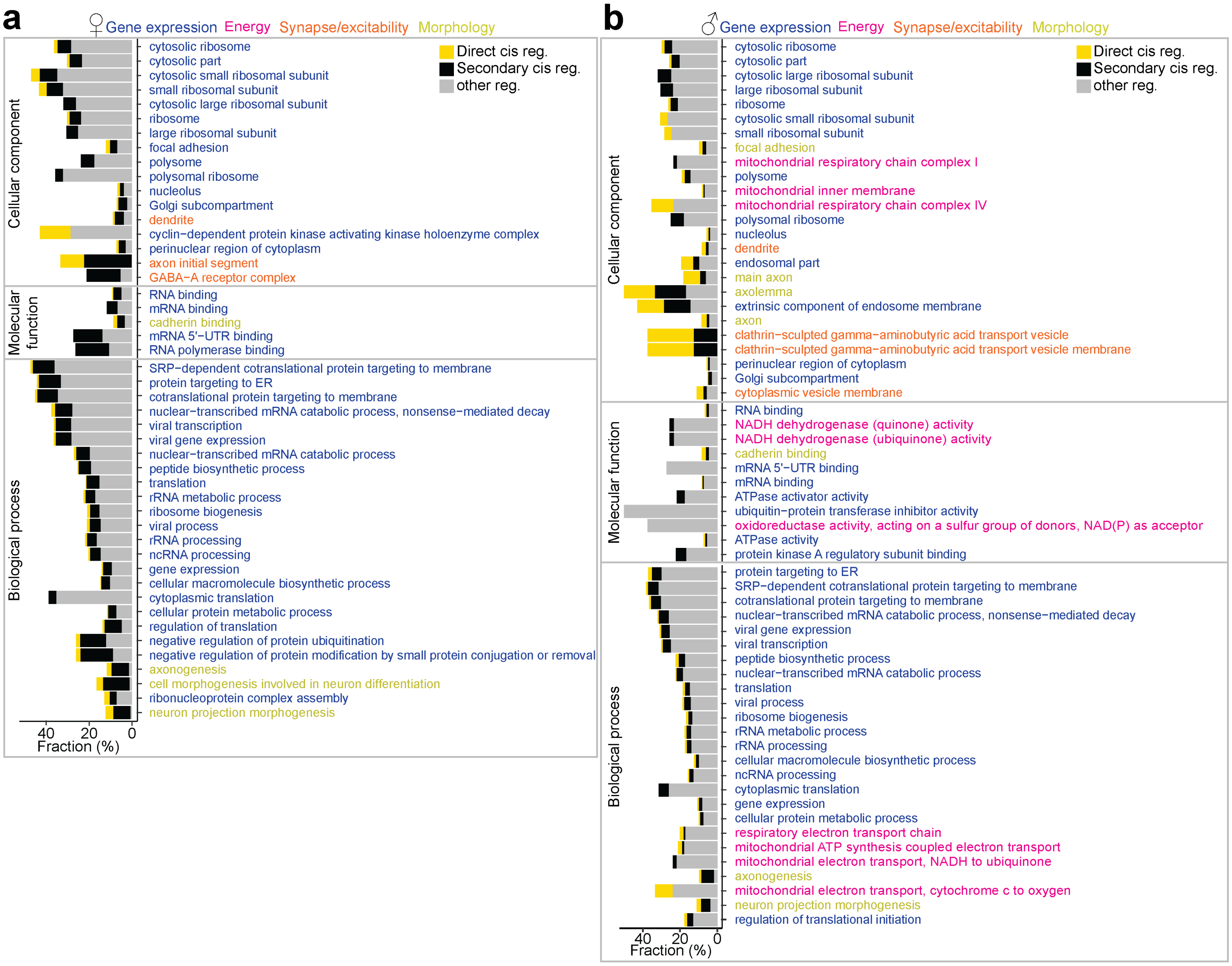

Supplementary Fig. 13: Supporting information for gene regulatory network analysis followed by cell-type specific deletion of Esr1, related to Fig. 8

a, b, Enriched Esr1-DEG gene ontology terms are placed alongside bar plots corresponding to gene expression percentage. Top 25 GO terms are shown and color-coded by gene category. Bars are fractionated into direct *Esr1*-regulated genes (yellow), secondary TF-regulated genes (black), and other genes regulated by Esr1-TFs and other TFs (grey). GO Biological Process, GO Molecular Function, and GO Cellular Component are referenced. **a:** females; **b:** males.

bioRxiv preprint doi: https://doi.org/10.1101/2025.02.26.640339; this version posted February 26, 2025. The copyright holder for this preprint (which was not certified by peer review) is the author/funder, who has granted bioRxiv a license to display the preprint in perpetuity. It is made available under aCC-BY 4.0 International license.

Supplementary Table 1. Data table showing quality of sequenced libraries and corresponding scRNAseq data from samples used in this study.

Supplementary Table 2. Data table showing the marker genes of neuronal clusters, related to **Fig. 2**.

Supplementary Table 3. Data table showing the DEGs in the pseudobulk populations between groups, related to **Supplementary Fig. 3**.

Supplementary Table 4. Data table showing the HA-DEGs in each Vgat+ cluster, related to **Supplementary Fig. 3**.

Supplementary Table 5. Data table showing the HA-DEGs in the Vgat+Esr1+ population, related to **Supplementary Fig. 3**.

Supplementary Table 6. Data table showing co-expression scores between transcription factors and adolescent gene programs or sexually dimorphic programs related to **Fig. 2 and 5**.

Supplementary Table 7. Data table showing the marker genes in the clusters associated with mating, related to **Fig. 2 and Supplementary Fig. 4**.

Supplementary Table 8. Data table showing the genes enriched in later trajectories or branch-DEGs in the Vgat+Esr1+ population, related to **Fig. 3 and Supplementary Fig. 5**.

Supplementary Table 9. Data table showing the Esr1-DEGs in the Vgat+Esr1+ or Vgat+/HormoneR^{low} populations, related to **Fig. 7**.

Supplementary Table 10. Data table showing the genes enriched in later trajectories or branch-DEGs in the Vgat+Esr1+ population, related to **Fig. 7** and **Supplementary Fig. 12**.

Supplementary Table 11. Data table showing the sexually dimorphic genes in the Vgat+Esr1+ population, related to **Fig. 7**.

Supplementary Table 12. *In situ* hybridization probe list.
